# Construction and content validation of a measurement tool to evaluate person-centered therapeutic relationships in physiotherapy services

**DOI:** 10.1371/journal.pone.0228916

**Published:** 2020-03-02

**Authors:** O. Rodríguez Nogueira, J. Botella-Rico, M. C. Martínez González, M. Leal Clavel, J Morera-Balaguer, A. R. Moreno-Poyato

**Affiliations:** 1 University of León, Health Sciences School, Nursing and Physical Therapy Department, Ponferrada León, Spain; 2 Universidad Cardenal Herrera-CEU, CEU Universities, Physical Therapy Department, Plaza Reyes Católicos, Elche, Alicante, Spain; 3 Universidad Cardenal Herrera-CEU, CEU Universities, Medicine Department, Plaza Reyes Católicos, Elche, Alicante, Spain; 4 Universidad Cardenal Herrera-CEU, CEU Universities, Nursing Department, Plaza Reyes Católicos, Elche, Alicante, Spain; 5 Universitat de Barcelona, Escola d'Infermeria, Departament d'Infermeria de Salut Pública, Salut Mental i MaternoInfantil, Facultat de Medicina i Ciències de la Salut, Campus Bellvitge Pavelló de Govern, c/ Feixa Llarga, L'Hospitalet de Llobregat Barcelona, Spain; Chinese Academy of Medical Sciences and Peking Union Medical College, CHINA

## Abstract

**Objectives:**

This study sought to develop a tool for evaluating person-centered therapeutic relationships within physiotherapy services, and to examine the content validity of the same.

**Methods:**

A mixed qualitative and quantitative study was performed in three distinct phases: 1) the items were generated based on a literature review and a content analysis of focus groups of patients and physiotherapists; 2) an e-Delphi survey process was performed based on three rounds to select and refine the proposed questionnaire; 3) two rounds of cognitive interviews were conducted to evaluate the comprehension of items, the clarity of language and the appropriateness and relevance of content.

**Results:**

Thirty-one items were generated based on the seven domains identified after the analysis of four focus groups of physiotherapists and four patient focus groups. Nine experts participated in the e-Delphi survey. Fifty-five patients participated in the two rounds of the cognitive pre-tests. Participating patients were from public and private physical therapy services. Based on the participants’ suggestions, four items were removed, and four were added, whereas 16 were reworded.

**Conclusions:**

The final tool comprised 31 items divided into seven domains. The response format was based on a 5-point Likert frequency scale. The response options ranged from “strongly agree” to “strongly disagree”.

## Introduction

Many professions recognize person-centered care (PCC) as being both a standard of quality [1], and a primary goal in itself [2]. In addition, PCC is understood as being a moral philosophy for health professionals seeking to provide the highest quality health care [3]. Despite the significance of PCC as an approach to care, there is no clear consensus regarding the definition of the same, nor its underlying dimensions [1,4–9]. Thus, a number of definitions are found for PCC and its components. According to the scientific literature reviewed, the therapeutic relationship established between the professional and the person receiving care is an inherent construct in the definition of PCC, understood as one of the fundamental factors underlying care. To illustrate this, the following different definitions of PCC are noted: “the interactions and alliance between the health professional and the patient, based on communication, health promotion and healthy lifestyles”; concern for providing individualized treatments, respecting people’s rights and the construction of a therapeutic relationship based on understanding and mutual trust [10]; a construct based on at least three different and important domains: communication, collaboration and health promotion [11]; the creation of a therapeutic narrative between the professional and the person, based on mutual trust, understanding, and sharing of knowledge [12]; a holistic approach for providing respectful and individualized care, enabling negotiated care, and offering choices within a therapeutic relationship, facilitating the patient’s involvement in health decisions as far as the person wishes to do so [13]. The model by Mead & Bower, probably the most frequently used for defining PCC, is based on five dimensions [14]: 1) the biopsychosocial perspective; 2) the “patient-as-person”; 3) sharing power and responsibility; 4) the therapeutic alliance; and 5) the “doctor-as person”, establishing the relationship between the professional and the person, as being key for development [4].

In addition, several authors have described certain characteristics of the therapeutic relationship as a means for establishing PCC. Thus, Constand et al [15] speak of communication and partnership; Zimmermann et al [9] highlight interactions and relationships, considering it important for professionals to be friendly and attentive; Hobbs [8] refers to “therapeutic engagement” and trust; Castro et al [4] underline empathy, trust, and individualized treatment; Rathert et al [5] establish the importance of respect, information, education, communication and emotional support; Wijma et al [6] mention trust, verbal communication adapted to the patient, nonverbal communication, and active listening; Scholl et al [16] highlight the importance of a reciprocal relationship characterized by constancy, trust, connection, mutual care, mutual knowledge, construction of a positive relationship and mutual understanding of roles and responsibilities.

Physiotherapy is adopting a biopsychosocial model [17], acknowledging that individual experiences, such as the social, cultural, psychological and contextual factors of a person exercise a strong influence on pathology and recovery [18]. Under this new paradigm, the establishment of PCC and a relationship between physiotherapists and those who receive care appear to be key for therapeutic success [19–21]. Indeed, there is a strong consensus among health professionals who believe quality of care directly depends on the establishment of a therapeutic relationship based on the individual [21,22].

Despite this, the PCC concept in physiotherapy is still poorly understood [6], and there is a lack of means for evaluating the established therapeutic relationship [21,23,24]. To construct an instrument that measures patient perceptions, content validity is the most important measurement property [25]. It is the degree to which the content of the instrument adequately reflects the construct to be measured in terms of relevance, clarity and coherence [26]. In order to obtain a degree of agreement regarding these three characteristics, the Delphi methodology is used, where a panel of experts, through several rounds of consultation, reach a previously established level of consensus [27,28].

Considering the need to establish the model of PCC in the field of physiotherapy and the importance of the person-centered therapeutic relationship within this model, this study sought to design an assessment tool and examine its content validity.

## Material and methods

**Fig 1:**
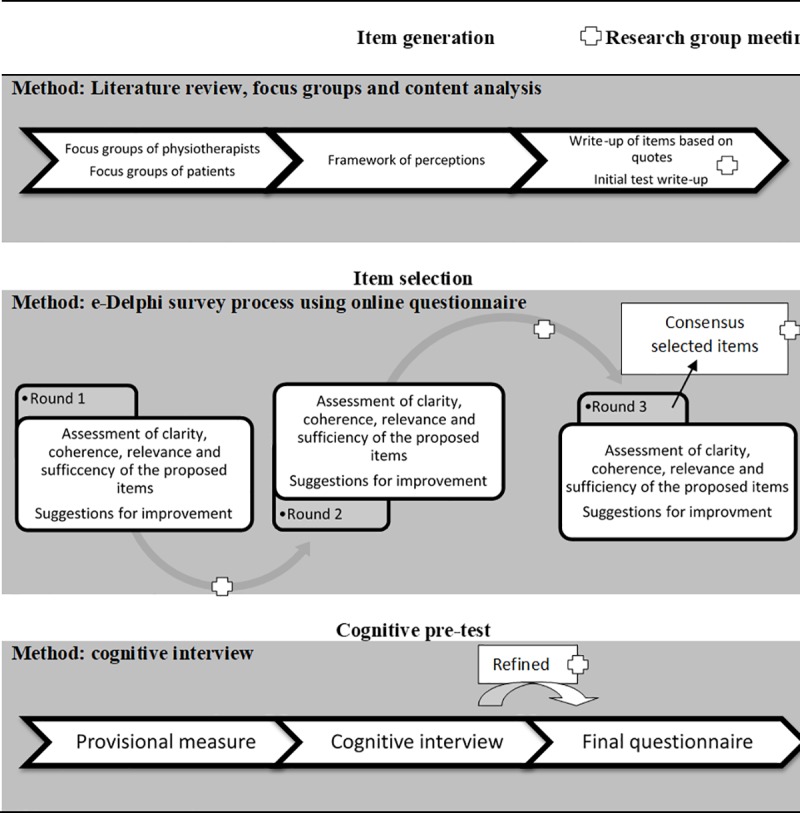
Research stage.

We used a mixed qualitative and quantitative study design. As depicted in Fig 1, our collaborative team of researchers (ORN, JMB, JBR, MCMG, MLC) conducted this study in three stages: 1) item generation, 2) item selection, and 3) pretesting of the questionnaire.

### Stage 1. Item generation

The items were generated via a review and analysis of the literature, based on two constructs: *person centered care and person-centered therapeutic relationship*. These findings enabled us to extract the components of each construct and create a question guide to explore the barriers and facilitators for the establishment of a person-centered relationship in physiotherapy services. This stage was performed via a qualitative study based on focus groups involving physiotherapists [23] and patients of physiotherapy services [29]. The findings from this study were used to develop a conceptual framework comprised of domains and subdomains.

The questionnaire items were created based on the identified subdomains. Thus, each member of the research team independently proposed several items for each subdomain [30]. The wording of the items was based on the quotes of the physiotherapists and patients who participated in the previous qualitative study [30,31].

Subsequently, two members of the research team (ORN, JMB) revised the items, selecting and modifying those which best suited the domains that they sought to improve, in order to enhance understanding of the same, avoid redundancy, maintain the conceptual framework and ensure conceptual coherence with the literature. To resolve any discrepancies between the two members of the research team (ORN, JMB), an independent researcher trained in therapeutic relations and in the study method was consulted (ARMP), who acted as reviewer. Through discussions and consensus directed by (ARMP), the advantages and disadvantages of each of the modified items were analyzed and, based on the same, an informed decision was made [32,33]. The items that were ultimately selected formed part of the initial questionnaire during the subsequent phase.

### Stage 2. Item selection

We conducted a three-round modified Delphi survey process via email correspondence to select and refine the proposed questionnaire (Fig 1). The purpose of the e-Delphi survey was to reach a consensus among the target users regarding clarity (ease of understanding), coherence (logical relation with the dimension or indicator that is being measured), and relevance (it is essential or important and therefore it must be included) of the items, as well as whether the items that belong to each dimension were sufficient in order to define and evaluate the dimension, or whether any aspect was overlooked [34].

#### Settings and participants

Theoretical sampling was used for the selection of participants in this study. This approach enables the selection of participants according to their relationship with the study phenomenon and following a criteria of suitability [35]. The chosen criteria were: 1) Health professionals interested in the patient-centered therapeutic relationship; 2) With knowledge and experience on this subject; 3) Who had demonstrated their capacity to theorize about the chosen subject, via research projects, theses, articles, communications, etc. during the previous three years [36,37].

A heterogeneous sample was sought in relation to the participants’ profession and age. Several authors have highlighted that a heterogeneous sample produces a higher proportion of quality responses compared to a homogenous group, as there is a certain diversity of points of view, generated by greater interest and reflection [38].

Recruitment took place by email and/or telephone. To do so, we revised which Spanish authors had published anything related with the subject under study during the previous three years, or who were presently conducting research on the subject. Special care was made to ensure that the study participants and their names remained anonymous, and we attempted to avoid recruiting participants belonging from institutions with which we have ties.

Each prospective participant was sent a letter with information on the study and a description of the Delphi process, as well as an informed consent form. Once the informed consent was signed and returned, participants were sent a questionnaire with the preselected items. To ensure that the participants were aware of the meaning of each dimension and sub dimension, we included a document with references to support the same.

#### Data collection

During the first round of the survey, the participants were asked to: 1) express their degree of agreement regarding clarity, coherence, and relevance for each of the items (1–4; 1 = strongly disagree, 4 = strongly agree); 2) if participants responded anything other than 4 = strongly agree, they were asked to justify their response, explaining how exactly they considered that item had failed, and providing optional recommendations for the improvement of the same; 3) lastly, considering the criteria of sufficiency, participants were asked to suggest new items for the subdomains treated, or for creating a new subdomain, if they considered this to be necessary.

After two members of the team (ORN, JMB) reviewed the participants’ responses, the questionnaire was sent to the participants, once again, with the modified items, an anonymous summary of the responses of all participants, and the mean results of each item. The participants were asked to repeat the same process considering all items.

After the second round, the questionnaire was revised once again by two members of the team (ORN, JMB) and, once more, a report was sent to all participants who were asked to repeat steps one and two.

Across the three rounds, we applied the same quantitative selection criteria for each item: 1) a mean score of ≥ 3.25 for degree of agreement, and 2) a rating of 3 or higher for degree of agreement among ≥ 70% of the participants in the Delphi survey [36]. Furthermore, we considered the recommendations made by the participants. Thus, when an item received new suggestions regarding wording, these suggestions were included and voted on in the following round. An item passed the selection stage when criteria one and two were fulfilled. If one of the two criteria was not fulfilled, a new wording was added, based on the participants’ recommendations. Likewise, if a new item appeared, this was subsequently voted upon.

### Stage 3. Pretesting of questionnaire

With the provisional questionnaire obtained after the performance of the Delphi survey, we performed a cognitive pre-test, with the following objectives: 1) To evaluate the understanding of the items and of the response options; 2) To evaluate the clarity of the language and format; 3) To evaluate the appropriateness and relevance of the content and the possible lack of aspects that were not initially considered; 4) To review any problems related to the order of questions or any interactions among the same; 5) To examine the perception of length or overall burden of the assessment tool.

Individual interviews were conducted using the *probing based* paradigm, in which the interviewer proactively guides the interaction, asking questions and using probing questions [39]. For this purpose, retrospective probing was used [39], where the participant responds to the complete questionnaire after which the interview takes place. Some authors consider that this is the weakest approach, because the participant may have forgotten what they were thinking when they responded to the question [40]. However, in this study, it was adapted to the circumstances in which the tool was to be applied: i.e. self-administration.

#### Settings and participants

The participants in this phase were patients with similar characteristics to those to whom the definitive tool was to be applied. For this, we established the following selection criteria: 1) patients over 18 years old; 2) who had received, at least, 15 physiotherapy treatment sessions; 3) without any cognitive impairments and comprehension difficulties.

The participants were recruited from two hospitals within the Spanish public health system (Madrid, A Coruña) and four private physiotherapy centers (Madrid, Orense, Elche) using convenience sampling methods. The physiotherapists from the centers where the respective patients received care were responsible for selecting and inviting participants. These health professionals participated voluntarily in the research and were previously informed of the study aims and the inclusion criteria for the patients during a designated meeting.

During the interviews, the researcher began by informing the participant of the study aim and provided an informed consent for the participant to sign. The participant subsequently completed the questionnaire. The participant was informed beforehand not to ask the researcher the meaning of any question. Should any participant have a query, they were asked to write the same in a blank text box included in the questionnaire. Once the questionnaire was completed, the researcher asked the participant about the meaning given to each item. This was done using several strategies, such as asking what the person had understood from the question or asking the participant to restate the questions using different wording. Finally, the participants were asked if they considered that any of the questions were inappropriate or unnecessary, whether they felt any item was missing, and to assess the difficulty of the questionnaire on a scale of 0 to 10 (0 = no difficulty; 10 = very difficult). In addition, they were asked whether they considered that the length of the questionnaire was appropriate or too long. The time that each participant took to complete the questionnaire was recorded, as an indirect measure of the difficulty of the same.

Four members of the research team conducted the interviews (ORN, JMB, JBR, MLC). Several interviewers were used, rather than a single interviewer as, although theoretically, the participants responses should be independent of the interviewer [41], there is evidence suggesting that the personality of the interviewer may influence the participants’ responses [41,42]. All the interviewers had training and experience in conducting cognitive interviews.

#### Data analysis

All the interviews were transcribed verbatim. Two researchers (ORN and JMB) analyzed the participants’ responses and coded any possible problems. For this purpose, a coding system was created, which corresponded with the four stages of the question-response process of CASM (Cognitive Aspects of survey Methodology)[43], adding a category related to instrument logic (S2 Table). A list was created with the items, the potential problems associated to each item, the proposals for changes, suggestions for new items, changes in the order, the format and the instructions, and data on the perception of difficulty and duration of the questionnaire. This list was presented to the entire research team. Subsequently, the research team met, discussed, and reached an agreement by consensus on whether to keep, modify, or remove each potentially defective item. Any potential problems were addressed from both a quantitative point of view (items with a frequency of acceptance below 85% required revision)[44] as well as a qualitative point of view. This collaborative approach sought to eliminate the potential bias of a single researcher’s perspective.

#### Ethical considerations

Each participant in the item generation phase granted consent. Participants provided informed written consent and indicated whether they wanted to be explicitly acknowledged in this paper. This study was approved by the Ethics Committee of the Cardenal Herrera CEU University, the ethical committees of the 12 de Octubre Hospital of Madrid and the General University Hospital of A Coruña.

## Results

### Item generation

Initially, 215 items were generated, based on the seven domains and 28 subdomains identified in the previous qualitative study (Table 1) [23,29].

**Table 1:** Conceptual framework of domains and subdomains.

**1. Personal characteristics of the professional**	1.1-Motivating and encouraging the involvement of the patient in the process based on a positive attitude 1.2- Perception of security, trust in oneself 1.3- The physiotherapist shows empathy towards the patient 1.4 –Authenticity of the physiotherapist towards the patient 1.5- Unconditional acceptance
**2. Communication capacities of the professional**	2.1 –Congruence between verbal and non-verbal communication 2.2- Non-verbal communication *2*.*2*.*1*: *tone and volume* *2*.*2*.*2*: *gaze* 2.3- Active listening skills 2.4- Verbal communication 2.5- Assertiveness
**3. Professional aspects**	3.1- Skill, competence, technical experience and knowledge 3.2- Professionality 3.3- Physiotherapist as educator 3.4- Follow-up of home prescriptions
**4. Relational aspects**	4.1- Affinity with the physiotherapist 4.2- Care 4.3- warmth (sensitive, kind, affectionate) 4.4- Close attitude 4.5- Displaying interest and involvement in the patient’s problem 4.6- Emotional support
**5. Personalized therapy**	5.1- Psycho-social-cultural sensitivity 5.2- Sensitivity to changes in the patient’s status
**6. Partnership**	6.1- Mutual trust 6.2- Mutual respect 6.3- Collaboration/active involvement *6*.*3*.*1*: *objectives and treatment* *6*.*3*.*2*: *how to treat problems*
**7. Environment**	7.1- Perception of coordination in the communication between the physiotherapist and other professionals 7.2- Perception of the physiotherapist as having professional autonomy 7.3- Physical space allowing privacy

After revising the proposed items, 184 items were removed as these were either considered redundant, or they clearly failed to represent the subdomain or because their wording could be improved. Of the 31 remaining items, the wording of 11 items was slightly modified. These items were assigned to the different subdomains of our previous conceptual framework, as follows: personal characteristics of the professional, (n = 6), capacity of communication (n = 6), professional aspects (n = 4), relational aspects (n = 6), personalized therapy (n = 2), partnership (n = 4), and environment (n = 3)

### Item selection

Of the 11 participants contacted for participation in the Delphi survey, two failed to respond. The nine remaining participants participated in the three rounds. Table 2 displays the sociodemographic characteristics of the nine participants in the Delphi survey. All had professional experience related with the survey theme or they were, or had been, involved in research projects related with the same.

**Table 2:** Sociodemographic data of Delphi participants.

Gender	n %
Male	7 77.77%
Female	2 22.22%
**Age (years) n %**	
35–45	5 55.55
46–55	1 11.11
56–65	2 22.22
66–75	1 11.11
Mean (years) 49.66	
**Studies n %**	
Physiotherapist	3 33.33
Psychologist	6 66.66
Nurse	3 33.33
Doctor	7 77.77
Professional profile n %	
University professor	7 77.77
Clinician	7 77.77
Researcher	9 100

Round 1 of the Delphi Survey lasted 3.5 months. As a result, 30 items (96.77%) fulfilled at least one of the two quantitative selection criteria, whereas one item failed to fulfill any criteria and was, therefore, removed. Four items did not fulfill one of the two selection criteria, therefore they were reformulated following the suggestions provided by the participants. Among the items that fulfilled the two selection criteria, in many of these, the participants made suggestions to improve the wording (between two and five suggestions), and provided comments about the item (between one and five). For example, in the case of the item *The treatment from my physiotherapist makes me feel better*, which was scored ≥ 3 by 100% of the participants, obtaining a mean score of 3.89, participants suggested the item be reworded as *The treatment from my physiotherapist makes me feel better emotionally*. Likewise, the item *You agree on the therapeutic objectives and the treatment*, which had fulfilled both criteria, was suggested to be reformulated as *You make a joint agreement on the therapeutic objectives and the treatment*. After reviewing the survey responses, the comments were discussed, together with the best proposals for the wording of the items and a new wording for the questionnaire was agreed by consensus, which included changes in 25 items. Likewise, one new item was included as proposed by participants. Finally, 31 items were included in the questionnaire presented in round two.

Round two lasted two months. Thirty items (96.77%) fulfilled the two quantitative selection criteria. One item, *do you feel that what you say is important for him/her and he/she tries to understand you*? changed from a score of 88.8% and 3.56 to 77.7% and 3.22, without fulfilling one of the criteria. The item *I believe that my physiotherapist and I have connected*, although it fulfilled both criteria, received a lower score compared to round one. These two items were reformulated based on the suggestions made by participants. This data is displayed in S1 Table.

In the case of the item *does he/she know how to tell you what he/she needs to say*, *clearly and firmly*, *without making you feel bad*?, which sought to measure assertiveness, 88.8% of participants evaluated this item with ≥ 3 and a mean score of 3.56, and one participant commented that it failed to include all the domains of assertiveness, whereas another participant proposed to change it to *does he/she give you the necessary information*, *clearly and firmly*, *without making you feel bad*?. For the item, *after explaining exercises or health advice*, *later asks you about these and goes over them if necessary*, 100% of participants scored it ≥ 3, obtaining a mean score of 3.67, which sought to measure the follow-up of home prescriptions. One participant commented that the term “care advice” could be in conflict with nursing competences, and proposed replacing the term with *health advice*.

After analyzing and discussing these four cases, a third round took place with all items. This third round lasted three weeks, during which the scores for the modified items fulfilled the selection criteria. The two items which had received lower scores in the second round attained a higher score and the participants did not provide any new suggestions. Therefore, these items were included in the final questionnaire.

Finally, a questionnaire was designed, based on 31 items, which was used for the subsequent stage.

### Pretesting of questionnaire

Two rounds of cognitive interviews were performed with 55 participants (n = 45 in the first round and n = 10 in the second round).

The duration of each interview was between 24 and 66 minutes. The mean time that participants took to complete the questionnaire was 6 minutes 40 seconds (3’ 2’” the fastest and 15 minutes the slowest). The perceived length of the same was deemed appropriate for most participants (88%). The mean perceived difficulty of the questionnaire was 2 (0 = very easy; 10 = very difficult).

The sociodemographic characteristics of the participants in both rounds are displayed in Table 3.

**Table 3:** Sociodemographic characteristics of participants’ pre-cognitive test.

Sex n %	
Men	14 25.4
Women	41 74.5
**Age (years) n %**	
18–28	2 3.6
29–38	5 9.1
39–48	8 14.5
49–58	11 20
59–68	19 34.5
69–78	7 12.7
79–88	3 5.4
Mean (years)	56.66
**Level of studies n %**	
Primary	14 25.4
Secondary	12 21.8
University	29 52.7
**Hospital type n %**	
Public	24 43.6
Private	31 56.4
**Pathology n %**	
Low back pain	12 21.8
Hip replacement	3 5.4
Neck pain	11 20
Bone fracture	12 21.8
Tongue cancer	1 1.8
Congenital spine malformation	1 1.8
Shoulder tendinopathy	8 14.5
COPD	4 7.3
Meniscus surgery	1 1.8
Guillain Barre	1 1.8
Polytrauma	1 1.8

The qualitative analysis of the interviews conducted during the first round revealed potential problems affecting 30 items, these concerned the response scale, the format and the structure of the questionnaire. Thus, 239 potential problems were detected in the first round, and 15 in the second round (the results of the cognitive pretest can be consulted in Table 4).

**Table 4:** Results of the pretesting of questionnaire.

Item	Acceptance item 1^st^/2^nd^ round	Nº of problems per round	Examples of potential problems	Suggestions	CASM category^a^	Final decision
General instructions	*N/A*	*0/0*		*Include “think and mark the response category which best describes your experience”*		*Edited*
Instructions for each item category	*N/A*	*0/0*		*Remove the sentence regarding context*		*The sentence providing context is removed*
1.1. I believe that my physiotherapist and I have connected	*89%/ 100%*	*5/0*	*They fail to understand the question*. *She feels that she should not respond because it’s her fault that they do not connect*. *A response option is missing (intermediate point)*	*Change the statement to "My physiotherapist and I understand each other"*	*CP (2/0)* *JD (1/0)* *RP (1/0)*	*Maintained*
1.2. I feel that my physiotherapist provides me with the best possible care and attention	93%/ 100%	3/0	*Considers that she cannot respond because it’s her fault that they cannot provide her with the best care* *A response option is missing (intermediate point)*		*JD (1/0)* *RP (2/0)*	*Maintained*
1.3. My physiotherapist is kind towards me	98%/100%	1/0			*RP (1/0)*	*Maintained*
1.4. I think that my physiotherapist is an accessible person.	96%/100%	2/0	*Does not understand what accessible means*		*CP (1/0)* *RP (1/0)*	*Maintained*
1.5. My physiotherapist is interested and concerned about my problem.	98%/100%	1/0	*A response option is missing (intermediate point)*		*RP (1/0)*	*Maintained*
1.6. The treatment from my physiotherapist makes me feel better emotionally.	93%/100%	3/0	*She considers that she shouldn’t respond because it’s her fault that they cannot make her feel better emotionally*. *A response option is missing (intermediate point)*		*JD (1/0)* *RP (2/0)*	*Maintained*
2.1 That your physiotherapist is interested in how you are as a person and treats you individually.	*96%*/100%	*2/0*	*Confusion with other characteristics*: *openness*, *sincerity …*		*CP (1/0)* *RP (1/0)*	*Maintained*
2.2. our physiotherapist identifies your physical and/or emotional status and adjusts the treatment according to the same.	84%/90%	7/1	*Only understands that the appropriate treatment is applied*. *Feels unable to respond because there is no way to know if the physiotherapist realizes this*. *Entails two questions* *Two similar to 2*.*1*	Considers that the word “emotional” should not be used.	*UND*. *(4/1)* *JD (1/0)* *LG (2/0)*	*Maintained*
3.1. There is mutual trust	94%/100%	3/0	*Does not understand the question (and doesn’t respond)* *Considers that he should not respond because it is something that depends on the patient*. *Too similar to 2*		*UND*. *(1/0)* *JD (1/0)* *LG (1/0)*	*Maintained*
3.2. There is a relationship based on respect.	100%/100%	0/0				*Maintained*
3.3. You make a joint agreement on the therapeutic objectives and the treatment.	*42%*	*26*	*Does not understand the term “therapeutic objective”* *Considers that the patient and the physiotherapist don’t have to agree on anything*		*UND*. *(15)* *LG (1)* *JD (10/1)*	*Removed*
**New item:** *My physiotherapist and I agree on what I want to achieve from the physiotherapy treatment*.	*90%*	*1*	*Considers that the physiotherapist is the one who must know*		*JD (1)*	*Maintained*
**New item:** *My physiotherapist and I agree on which treatment to follow*.	*90%*	*1*	*Considers that the physiotherapist is the one who must decide*		*JD (1)*	*Maintained*
3.4 You collaborate to resolve together the problems that may arise during your rehabilitation	62%	17	*Believe that we are asking them whether they follow the physiotherapist’s instructions*. *Do not consider that there should be a collaboration between the patient and the physiotherapist*. *Too similar to 3*.*3*		*CP (10)* *JD (1)* *RP (3)* *LG (3)*	*Removed*
4.1. knows perfectly well what he/she has to do?	89%/100%	5/0	*Understand that we are referring to whether the patient understands what the physiotherapist tells them to do*		*CP (5/0)*	*Maintained*
4.2. acts in the best possible way to improve your problem.	91%/100%	4/0	*Responds only thinking of treatment results*		*CP (4/0)*	*My physiotherapist performs his/her work with seriousness and honesty*.
4.3… informs you of your problem and the physiotherapy treatment options	*76%/* *(first item)* *76%/ (second item)*	*11*	*Feels that it is exclusively up to the physiotherapist to apply whatever treatment the therapist considers* *Entails two questions*		*CP (5/0)* *JD (5/0)* *LG (1/0)*	*Removed*
**New item:** *The physiotherapist informs me of my health problem*	*100%*	*0*				Maintained
**New item:** *The physiotherapist informs me of the physiotherapy treatment options for my problem*	*96%*	*1*	*Believes that it is an exclusive function of the physiotherapist to apply whatever treatment esteemed appropriate*.		*JD (1)*	Maintained
4.4…. after explaining exercises or health advice, later asks you about these and goes over them if necessary?	93%/100%	3/0	*This question is not applicable because the physiotherapist has not explained any exercises*. *Entails two questions*		*CP (2/0)* *RP (1/0)*	*When my physiotherapist explains exercises or health advice to me*, *he/she then asks me about these and goes over them if necessary*.
5.1… do you notice that his/her body gestures, gaze and words are clear and not contradictory?	87%/100%	6/0	*Understands that we are asking whether the patient understands what the physiotherapist says* *Too similar to 5*.*2*		*CP (4/0)* *RP (1/0)* *LG (1/0)*	*I feel that the words and gestures of my physiotherapist contradict each other*.
5.2… the expressions, tone and volume of the voice of your physiotherapist generate trust and proximity?	100%//100%	1/0	*Too similar to 5*.*1*		*LG (1/0)*	*The tone and volume of the voice of my physiotherapist generate trust*.
5.3… your therapist’s gaze generates confidence and ease	84%/90%	7/1	*Too similar to 5*.*1*	Change the order of items 5.1, 5.2 and 5.3, so they are not one after the other. A response option is missing (intermediate point) Unify items 5.1, 5.2 and 5.3	*CP (5/0)* *RP (1/0)* *LG (1/1)*	*My physiotherapist’s gaze generates trust*.
5.4… do you feel that what you say is important for him/her and he/she tries to understand you?	78%/90%	10/1	*Understands that it’s the patient who must understand what the physiotherapist says*		*CP (9/1)* *RP (1/0)*	*I feel that my physiotherapist is interested in what I say*.
5.5… does he/she speak to you in an easy and simple manner?	*98%*/100%	*1/0*	*Responds without UND*., *trying to manifest how satisfied she is with her physiotherapist*.		*RP (1/0)*	*My physiotherapist speaks to me in an easy and simple manner*.
5.6… is he/she interested in knowing whether you have understood what he/she says?	87%/100%	6	*Responds without UND*., *trying to manifest how satisfied she is with her physiotherapist*. *Feels this is already addressed in the previous item (4*.*4)*		*CP (1)* *LG (1)*	Removed
5.7… does he/she give you the necessary information, clearly and firmly, without making you feel bad?	93%/100%	3/0	*Does not understand the sentence “Without making you feel bad"* *There are several questions on the same item*. *Too similar to 5*.*6*		*CP (1/0)* *LG (2/0)*	*My physiotherapist knows how to express opinions opposed to mine*, *without making me feel bad*.
6.1… makes you believe in your capabilities to get ahead with your effort?	89%/100%	5/0	*Do not understand whether we refer to the patient’s effort or that of the physiotherapist*. *Does not consider that they have to respond because “that is not the role of the physiotherapist”*. *Too similar to 4*.*1*	*"He tries to infuse confidence in myself"*	*CP (3/0)* *JD (1/0)* *LG (1/0)*	*My physiotherapist makes me believe that I am able to get ahead with my own effort*.
6.2…conveys reassurance in what he/she tells you or says during the treatment process?	98%/100%	1/0	*Responds without UND*., *trying to manifest how satisfied she is with her physiotherapist*. .		*CP (1/0)*	*My physiotherapist makes me feel secure in what he says or does during the treatment process*.
6.3…understands how you feel and tries to put him/herself in your place?	91%/100%	4/0	*Incoherent response*: *“there has not been any opportunity”* *Considers that he/she shouldn’t respond because nobody can put themselves in someone else’s place*.		*CP (2/0)* *JD (1/0)* *RP (1/0)*	*My physiotherapist understands how I feel*
6.4.… appears natural, sincere and honest at all times?	*93%*/100%	*3/0*	*Considers that he/she shouldn’t respond because it isn’t possible to know this*.		*CP (2/0)* *JD (1/0)*	My physiotherapist appears natural, sincere and honest at all times
6.5… has made you feel judged at any time.	73%/90%	12/1	*Considers that this question is too difficult to respond to as it is too deep*.		*CP(10/0)* *JD (2/1)*	*I feel that my physiotherapist accepts me as I am*.
7.1. I observe a lack of communication or coordination among the team of professionals who attends me.	42%/90%	26/1	*The concept “team of professionals” is misunderstood*. *The concept “communication and coordination” is misunderstood*.	At times they are unable to respond because they are treated by a single physiotherapist	*CP(22/0)* *RP (3/0)* *JD (1/1)*	*I observe a lack of coordination between the team of professionals (physiotherapists*, *doctors*, *aids*, *administration staff*, *etc) who attend me*.
7.2. I feel that my therapist makes decisions independently regarding his/her treatment area	36%/90%	29/1	*The concept “treatment area” is misunderstood*. *Feels that this refers to independence toward the patient*. *Considers that he/she should not respond because it is clear that the physiotherapist is not independent*.		*CP(28/1)* *JD (1/0)*	*I feel that my physiotherapist has autonomy when making decisions about my treatment*.
7.3. I feel that the space where the therapy takes place provides me privacy.	87%/100%	6/0	*Understands that we are referring to data protection*. *Considers that he/she cannot respond because the space should provide safety and not privacy*		*CP (4/1)* *JD (1/1)* *RP (1/1)*	Maintained

^**a**^CP: comprehension JD: judgement RP: reporting UND: understanding LG: logic

Three people indicated that a central response option was lacking for the entire questionnaire. Comprehension problems affected 24 items in round one and one item in the second round. For example, in the item *do you feel that what you say is important for him/her and he/she tries to understand you*? some users were unable to understand the item, whereas other understood it with a different meaning to what was initially intended (assuming that the patient was the one who had to understand the physiotherapist). Eleven items presented instrument logic problems in round one, whereas in round 1, only one item was affected. For example, some participants said that the item *does he/she give you the necessary information*, *clearly and firmly*, *without making you feel bad*? contained several questions. Fourteen items presented judgement problems in the first round and two in the second round. For example, for item *understands how you feel and tries to put him/herself in your place*? one patient stated: “I understand the question, but I have no way of knowing”. Or the item *has made you feel judged at any time*?, for which two participants stated that it was “a very severe accusation”. Fifteen items presented reporting problems in the first round. For example, two participants told us that in item *The treatment from my physiotherapist makes me feel better emotionally*, a response option was missing (an intermediate score).

Several participants commented that the introductory sentence which was placed after the items to which they had to respond hampered the comprehensin of the same.

Finally, four items were removed, and 16 were reformulated according to the participants’ suggestions. For example, the item *makes you believe in your capabilities to get ahead with your effort*? was reworded as… *makes you believe that you are able to get ahead with your effort*? In addition, based on the participants’ proposals, four new items were added. Lastly, the format of the document was changed, as well as the order of 14 items, and the heading providing the context of the items was removed.

With the refined questionnaire, a second round of cognitive interviews was performed. In this second round, all the items fulfilled the quantitative acceptance criteria, no important potential problem was detected from the qualitative point of view and there were no new suggestions, neither were there any potential problems in the format of the document or with the order of the questions.

The final tool contains 31 items divided into seven domains. The response format is based on a 5-point Likert frequency scale. Response options range from “strongly agree” to “strongly disagree”. A subsequent study will calculate the psychometric properties of this tool.

## Discussion

The relationship that is established between professionals and patients is of vital importance for establishing person centered care; a priority for the reorganization of health care in the 21st century [6]. In this study, we present a process of construction and content validation of a questionnaire to measure the therapeutic patient centered relationship in physiotherapy. The content validity guarantees that the elements included in the tool reflect the construct of the same. To the best of our knowledge, no instrument of these characteristics exists in physiotherapy services. The most used instruments to evaluate this relationship are the Working Alliance Inventory (WAI) and The Helping Alliance Questionnaire Version Two (HAQ-II)[21,45]. Both measure the “therapeutic alliance” construct, which means collaboration, warmth and support between the client and the therapist [46]. However, this is only one part of the therapeutic relationship construct [47] necessary for the establishment of PCC[14], thus, a content validation of the remaining domains required for establishing a supportive PCC relationship is clearly called for.

The different processes used for the construction and validation of this questionnaire add strength to its content validity. Firstly, by basing this study on a previous review of the literature, one of the necessary requirements is fulfilled to ensure an adequate psychometric guarantee: the definition of the evaluated construct and its components[48].

One of the strengths of this study is that it considers the point of view of the patients who received the care. Considering that the opinions of the professionals and patients can differ in important elements [49], focus groups were conducted where, besides the perceptions of physiotherapists, [23], the perceptions of the 31 patients were gathered, which was key for establishing the seven domains of the instrument [29] and for generating the items of the same.

In the item generation phase, a method was used that with demonstrated its validity [30,31]. In addition, the team of researchers who participated in this phase were asked to try to create items using the vocabulary that had previously been analyzed in the qualitative study, in order to improve comprehension.

Once the items were generated, a Delphi methodology was used to assess clarity, coherence and relevance, as well as the sufficiency of the items belonging to each dimension to obtain measurement scores. The Delphi method is considered to be the method of choice for a structured group discussion with the objective of reaching a high group consensus [50] and has been used in various types of studies, including the creation and validation of questionnaires [51]. Among other characteristics, it is worth noting that this method allows experts to issue opinions blinded to the opinions of other experts in the first phase, and to achieve an anonymous consensus, avoiding a "leading effect" bias [50]. This is how this process was conducted, preventing experts from knowing the identity of the other experts who collaborated. The high rate of agreement achieved after the consultation rounds, and the fact that all dimensions and subdimensions were recognized as relevant, not including any more feedback by the experts, gives us the idea of the content validity of the instrument. We considered it relevant to indicate that the criteria for the selection of items in our study were similar [36] or even more severe than those commonly used in the literature. In addition, the introduction of a system that allowed participants to make qualitative contributions through open-ended responses improves the validation process.

Two rounds of cognitive pretesting were completed, the first, with 45 patients, and a second round with 10 patients, thus complying with the known sample size for conducting a cognitive pretest which is between five and 15, per round [42]. We were stricter than what the literature tends to advise with regards the review of items after a cognitive pretest as, even the items that were above what the literature suggests as being the percentage of acceptance for revising an item [44], we introduced minor changes based on the participants’ suggestions, when the research team considered that these suggestions would effectively improve the item. These changes were subsequently well received by the participants.

Our intention, among others, was for patients to evaluate both the understanding. of the items, as well as their relevance and a possible lack of important aspects not initially considered. The cognitive pre-test technique, by means of cognitive interviews, is a resource commonly used for this purpose, with good results [36]. This technique involves patients with similar characteristics to those to whom the final tool is addressed, providing us of an idea of the importance given to the patients’ perceptions in the drafting of a tool which intends to measure patient centered care. Thus, after the cognitive pre-test, 10 items were removed, and another 10 were added, as proposed by participants, while a further seven were partially reformulated. All of this was intended to improve the understanding. of the items, as there were no discrepancies regarding the relevance of the same.

One of the key factors for establishing the PCC is teamwork and the establishment of objectives and treatments based on the patient’s beliefs and interests [11–13]. To evaluate this, the questionnaire includes a partnership dimension, consisting of four items. One of these refers to reaching agreements for establishing treatment objectives: *My physiotherapist and I agree on what I want to achieve from the physiotherapy treatment*, while another refers to agreements concerning treatment plans: *My physiotherapist and I agree on which treatment to follow*. Some of the most relevant comments made by patients during the cognitive pre-test were that the weight of the decision relies on the physiotherapist or that the patient does what the physiotherapist tells them to do, and that the one who knows what to do is the physiotherapist, or that the patient just agrees to everything. The patients understood the meaning of the items but were not accustomed to establishing objectives based on their preferences and, even less, deciding on treatment options. Typically, they were only provided with information of their problem and the treatment that they were to receive, perceiving that when the physiotherapist tells them to do something, they must comply. This underlines the need to construct and validate the present assessment tool, in order to be able to evaluate the relationship competences of physiotherapists and to influence the education of professionals in order to establish PCC.

Regarding the Delphi methodology, it is important to highlight that this method allows experts to provide opinions blinded to the opinions of other experts during the first stage, and the achievement of an anonymous consensus, avoiding a “leader effect” bias [52]. These methods were used to avoid the experts knowing the identity of the other experts collaborating in the study. Concerning the number of experts necessary when using this methodology, there is no established consensus, although a number of experts of around 10 is usually considered sufficient to be considered valid [53,54]. In our case, the number of experts was nine, which we consider to be sufficient, considering the heterogeneity of the sample [55]. The high rate of agreement achieved after the consultation rounds, and the fact that all the domains and subdomains were recognized as being relevant, without suggestions for further items to be included on behalf of the experts, helps support the validity of the instrument content. Furthermore, the introduction of a system to enable participants to provide qualitative suggestions regarding the aspects studied (clarity, coherence, relevance and sufficiency), allowed participants to propose new items, which improves the process of validation.

This study presents a series of limitations. The most important is the non-inclusion of patients within the Delphi methodology. We feel that the therapeutic relationship is a construct for which its meaning and essence is still being explored [56]. Therefore, we preferred to gather an opinion regarding the domains found and whether the items expressed the subdomains which they hoped to measure by consulting experts on the subject.

In conclusion, we present the content validity of an assessment tool for examining the quality of the therapeutic person centered relationship in physiotherapy services. This tool comprises seven domains and 31 items. We consider that it is a useful and appropriate tool for studying PCC, and that it will be invaluable for facilitating the understanding. and establishment of PCC in physiotherapy services.

## Supporting information

S1 File Annex 2. (DOCX)

S2 File CUESTIONARIO (Spanish). (DOCX)

S3 File QUESTIONNAIRE. (DOCX)

S1 Table Delphi results. (PDF)

S2 Table Coding system for the analysis of cognitive interviews. (PDF)
