# Supplementary material for: Construction and content validation of a measurement tool to evaluate person-centered therapeutic relationships in physiotherapy services
Source: PLoS One. 2020 Mar 2;15(3):e0228916. doi: 10.1371/journal.pone.0228916 (PMC7051061; doi:10.1371/journal.pone.0228916)
Supplement: S2 File — (DOCX) [file pone.0228916.s002.docx]

CUESTIONARIO DE RELACIÓN TERAPÉUTICA CENTRADA EN LA PERSONA EN FISIOTERAPIA

Este cuestionario está dirigido a CONOCER LA RELACIÓN TERAPÉUTICA ENTRE LOS PACIENTES Y SUS FISIOTERAPEUTAS

Diversos estudios han demostrado su importancia en los procesos de recuperación del paciente

SU información nos puede AYUDAR a MEJORAR LA RELACIÓN TERAPÉUTICA

INSTRUCCIONES para rellenar el cuestionario:

## 1) A continuación aparece una lista de afirmaciones y preguntas acerca de sus experiencias personales con su fisioterapeuta. Piense y señale qué categoría de respuesta describe mejor su propia experiencia.

## 2) Lea bien las preguntas y las respuestas

## 3) NO DEJE preguntas SIN contestar.

3) *Señale así* ⌧ *las respuestas adecuadas* (hágalo con cuidado para no equivocarse).

**Indique su grado de acuerdo con respecto a las siguientes afirmaciones:**

*1. Creo que mi fisioterapeuta y yo hemos conectado.*

| Totalmente de acuerdo |  | De acuerdo |  | Ni de acuerdo ni en desacuerdo |  | En desacuerdo |  | Totalmente en  desacuerdo |
| --- | --- | --- | --- | --- | --- | --- | --- | --- |

*2. Siento que mi fisioterapeuta me proporciona el mejor cuidado y atención posibles.*

| Totalmente de acuerdo |  | De acuerdo |  | Ni de acuerdo ni en desacuerdo |  | En desacuerdo |  | Totalmente en  desacuerdo |
| --- | --- | --- | --- | --- | --- | --- | --- | --- |

*3. Mi fisioterapeuta es amable conmigo.*

| Totalmente de acuerdo |  | De acuerdo |  | Ni de acuerdo ni en desacuerdo |  | En desacuerdo |  | Totalmente en  desacuerdo |
| --- | --- | --- | --- | --- | --- | --- | --- | --- |

*4. Creo que mi fisioterapeuta es una persona accesible.*

| Totalmente de acuerdo |  | De acuerdo |  | Ni de acuerdo ni en desacuerdo |  | En desacuerdo |  | Totalmente en  desacuerdo |
| --- | --- | --- | --- | --- | --- | --- | --- | --- |

*5. Mi fisioterapeuta se interesa y preocupa por mi problema.*

| Totalmente de acuerdo |  | De acuerdo |  | Ni de acuerdo ni en desacuerdo |  | En desacuerdo |  | Totalmente en  desacuerdo |
| --- | --- | --- | --- | --- | --- | --- | --- | --- |

*6. El trato con mi fisioterapeuta me hace sentir mejor emocionalmente.*

| Totalmente de acuerdo |  | De acuerdo |  | Ni de acuerdo ni en desacuerdo |  | En desacuerdo |  | Totalmente en  desacuerdo |
| --- | --- | --- | --- | --- | --- | --- | --- | --- |

*7. Mi fisioterapeuta se interesa en cómo soy como persona y me trata de manera individual*

| Totalmente de acuerdo |  | De acuerdo |  | Ni de acuerdo ni en desacuerdo |  | En desacuerdo |  | Totalmente en  desacuerdo |
| --- | --- | --- | --- | --- | --- | --- | --- | --- |

*8. Mi fisioterapeuta identifica mi estado físico y/o emocional y ajusta el tratamiento en función del mismo*

| Totalmente de acuerdo |  | De acuerdo |  | Ni de acuerdo ni en desacuerdo |  | En desacuerdo |  | Totalmente en  desacuerdo |
| --- | --- | --- | --- | --- | --- | --- | --- | --- |

*9. Entre mi fisioterapeuta y yo existe confianza mutua.*

| Totalmente de acuerdo |  | De acuerdo |  | Ni de acuerdo ni en desacuerdo |  | En desacuerdo |  | Totalmente en  desacuerdo |
| --- | --- | --- | --- | --- | --- | --- | --- | --- |

*10. Entre mi fisioterapeuta y yo hay una relación basada en el respeto.*

| Totalmente de acuerdo |  | De acuerdo |  | Ni de acuerdo ni en desacuerdo |  | En desacuerdo |  | Totalmente en  desacuerdo |
| --- | --- | --- | --- | --- | --- | --- | --- | --- |

*11. Entre mi fisioterapeuta y yo nos ponemos de acuerdo sobre lo que yo quiero conseguir con el tratamiento de Fisioterapia.*

| Totalmente de acuerdo |  | De acuerdo |  | Ni de acuerdo ni en desacuerdo |  | En desacuerdo |  | Totalmente en  desacuerdo |
| --- | --- | --- | --- | --- | --- | --- | --- | --- |

*12. Mi fisioterapeuta y yo nos ponemos de acuerdo sobre el tratamiento a seguir*

| Totalmente de acuerdo |  | De acuerdo |  | Ni de acuerdo ni en desacuerdo |  | En desacuerdo |  | Totalmente en  desacuerdo |
| --- | --- | --- | --- | --- | --- | --- | --- | --- |

*13. Mi fisioterapeuta sabe perfectamente lo que tiene que hacer.*

| Totalmente de acuerdo |  | De acuerdo |  | Ni de acuerdo ni en desacuerdo |  | En desacuerdo |  | Totalmente en  desacuerdo |
| --- | --- | --- | --- | --- | --- | --- | --- | --- |

*14.*  *Mi fisioterapeuta* *realiza su trabajo con seriedad y honradez.*

| Totalmente de acuerdo |  | De acuerdo |  | Ni de acuerdo ni en desacuerdo |  | En desacuerdo |  | Totalmente en  desacuerdo |
| --- | --- | --- | --- | --- | --- | --- | --- | --- |

*15. Mi fisioterapeuta me informa sobre mi problema de salud.*

| Totalmente de acuerdo |  | De acuerdo |  | Ni de acuerdo ni en desacuerdo |  | En desacuerdo |  | Totalmente en  desacuerdo |
| --- | --- | --- | --- | --- | --- | --- | --- | --- |

*16. Mi fisioterapeuta me informa sobre las opciones de tratamiento de fisioterapia para mi problema.*

| Totalmente de acuerdo |  | De acuerdo |  | Ni de acuerdo ni en desacuerdo |  | En desacuerdo |  | Totalmente en  desacuerdo |
| --- | --- | --- | --- | --- | --- | --- | --- | --- |

*17. Cuando mi fisioterapeuta me explica ejercicios o consejos para mi salud, después me pregunta por ellos y los revisa si es necesario.*

| Totalmente en desacuerdo |  | En desacuerdo |  | Ni de acuerdo ni en desacuerdo |  | De  acuerdo |  | Totalmente de acuerdo |
| --- | --- | --- | --- | --- | --- | --- | --- | --- |

*18. Mi fisioterapeuta me hace creer que tengo la capacidad para salir adelante con mi propio esfuerzo.*

| Totalmente en desacuerdo |  | En desacuerdo |  | Ni de acuerdo ni en desacuerdo |  | De  acuerdo |  | Totalmente de acuerdo |
| --- | --- | --- | --- | --- | --- | --- | --- | --- |

*19. Mi fisioterapeuta me transmite seguridad en lo que dice o hace en el proceso de tratamiento.*

| Totalmente en desacuerdo |  | En desacuerdo |  | Ni de acuerdo ni en desacuerdo |  | De  acuerdo |  | Totalmente de acuerdo |
| --- | --- | --- | --- | --- | --- | --- | --- | --- |

*20. Mi fisioterapeuta comprende cómo me siento.*

| Totalmente en desacuerdo |  | En desacuerdo |  | Ni de acuerdo ni en desacuerdo |  | De  acuerdo |  | Totalmente de acuerdo |
| --- | --- | --- | --- | --- | --- | --- | --- | --- |

21. *Mi fisioterapeuta se muestra natural, sincero/a y honesto/a en todo momento.*

| Totalmente en desacuerdo |  | En desacuerdo |  | Ni de acuerdo ni en desacuerdo |  | De  acuerdo |  | Totalmente de acuerdo |
| --- | --- | --- | --- | --- | --- | --- | --- | --- |

*22. Siento que mi fisioterapeuta me acepta tal y como soy.*

| Totalmente en desacuerdo |  | En desacuerdo |  | Ni de acuerdo ni en desacuerdo |  | De  acuerdo |  | Totalmente de acuerdo |
| --- | --- | --- | --- | --- | --- | --- | --- | --- |

*23. Observo falta de coordinación entre el equipo de profesionales (fisioterapeutas, médicos, auxiliares, personal de administración, etc) que me atiende.*

(Atención: en caso de realizar el tratamiento de fisioterapia en un centro donde únicamente trabaja el fisioterapeuta marcar la casilla No procede)

| Totalmente en desacuerdo | En desacuerdo | Ni de acuerdo ni en desacuerdo | De  acuerdo | Totalmente de acuerdo |  | No procede |  |  |  | Totalmente en desacuerdo |  | En desacuerdo |  | Ni de acuerdo ni en desacuerdo |  | De  acuerdo |  | Totalmente de acuerdo |
| --- | --- | --- | --- | --- | --- | --- | --- | --- | --- | --- | --- | --- | --- | --- | --- | --- | --- | --- |

*24. Percibo que mi fisioterapeuta tiene autonomía a la hora de tomar decisiones sobre mi tratamiento.*

| Totalmente en desacuerdo |  | En desacuerdo |  | Ni de acuerdo ni en desacuerdo |  | De  acuerdo |  | Totalmente de acuerdo |
| --- | --- | --- | --- | --- | --- | --- | --- | --- |

*25. Siento que el espacio donde se realiza la terapia me proporciona intimidad.*

| Totalmente en desacuerdo |  | En desacuerdo |  | Ni de acuerdo ni en desacuerdo |  | De  acuerdo |  | Totalmente de acuerdo |
| --- | --- | --- | --- | --- | --- | --- | --- | --- |

*26. Siento que las palabras y los gestos de mi fisioterapeuta se contradicen.*

| Totalmente en desacuerdo |  | En desacuerdo |  | Ni de acuerdo ni en desacuerdo |  | De  acuerdo |  | Totalmente de acuerdo |
| --- | --- | --- | --- | --- | --- | --- | --- | --- |

*27. El tono y el volumen de voz de mi fisioterapeuta me generan confianza.*

| Totalmente en desacuerdo |  | En desacuerdo |  | Ni de acuerdo ni en desacuerdo |  | De  acuerdo |  | Totalmente de acuerdo |
| --- | --- | --- | --- | --- | --- | --- | --- | --- |

*28. La mirada de mi fisioterapeuta me genera confianza.*

| Totalmente en desacuerdo |  | En desacuerdo |  | Ni de acuerdo ni en desacuerdo |  | De  acuerdo |  | Totalmente de acuerdo |
| --- | --- | --- | --- | --- | --- | --- | --- | --- |

29. *Siento que mi fisioterapeuta se interesa por lo que le digo.*

| Totalmente en desacuerdo |  | En desacuerdo |  | Ni de acuerdo ni en desacuerdo |  | De  acuerdo |  | Totalmente de acuerdo |
| --- | --- | --- | --- | --- | --- | --- | --- | --- |

30. *Mi fisioterapeuta me habla de manera fácil y sencilla.*

| Totalmente en desacuerdo |  | En desacuerdo |  | Ni de acuerdo ni en desacuerdo |  | De  acuerdo |  | Totalmente de acuerdo |
| --- | --- | --- | --- | --- | --- | --- | --- | --- |

*31. Mi fisioterapeuta sabe expresar opiniones contrarias a las mías sin hacerme sentir mal.*

| Totalmente en desacuerdo |  | En desacuerdo |  | Ni de acuerdo ni en desacuerdo |  | De  acuerdo |  | Totalmente de acuerdo |
| --- | --- | --- | --- | --- | --- | --- | --- | --- |

**¡MUCHAS GRACIAS POR SU COLABORACIÓN!**
