# Supplementary material for: Construction and content validation of a measurement tool to evaluate person-centered therapeutic relationships in physiotherapy services
Source: PLoS One. 2020 Mar 2;15(3):e0228916. doi: 10.1371/journal.pone.0228916 (PMC7051061; doi:10.1371/journal.pone.0228916)
Supplement: S3 File — (DOCX) [file pone.0228916.s003.docx]

SCALE ON PERSON-CENTERED THERAPEUTIC RELATIONSHIPS IN PHYSIOTHERAPY

This questionnaire is directed at EXAMINING THE THERAPEUTIC RELATIONSHIP BETWEEN PATIENTS AND THEIR PHYSIOTHERAPISTS

Several studies have demonstrated the importance of this in the processes of patient improvement

YOUR information can HELP us to IMPROVE THE THERAPEUTIC RELATIONSHIP

INSTRUCTIONS for completing the questionnaire:

## 1) Below, is a list of statements and questions pertaining to your personal experiences with your physiotherapist. Reflect on each question and mark which response category best describes you own experience.

## 2) Please read carefully both the questions and the responses

## 3) DO NOT leave any questions UNRESPONDED.

3) *Mark the appropriate answers with a cross* ⌧ (do so carefully to avoid making mistakes).

**Indicate your level of agreement regarding the following affirmations:**

*1. I believe that my physiotherapist and I have connected.*

| Completely agree |  | Agree |  | Neither agree nor disagree |  | Disagree |  | Completely disagree |
| --- | --- | --- | --- | --- | --- | --- | --- | --- |

*2. I feel that my physiotherapist provides me with the best possible care and attention.*

| Completely agree |  | Agree |  | Neither agree nor disagree |  | Disagree |  | Completely disagree |
| --- | --- | --- | --- | --- | --- | --- | --- | --- |

*3. My physiotherapist is kind towards me.*

| Completely agree |  | Agree |  | Neither agree nor disagree |  | Disagree |  | Completely disagree |
| --- | --- | --- | --- | --- | --- | --- | --- | --- |

*4. I think that my physiotherapist is an accessible person.*

| Completely agree |  | Agree |  | Neither agree nor disagree |  | Disagree |  | Completely disagree |
| --- | --- | --- | --- | --- | --- | --- | --- | --- |

*5. My physiotherapist is interested and concerned about my problem.*

| Completely agree |  | Agree |  | Neither agree nor disagree |  | Disagree |  | Completely disagree |
| --- | --- | --- | --- | --- | --- | --- | --- | --- |

*6. The treatment from my physiotherapist makes me feel better emotionally.*

| Completely agree |  | Agree |  | Neither agree nor disagree |  | Disagree |  | Completely disagree |
| --- | --- | --- | --- | --- | --- | --- | --- | --- |

*7. My physiotherapist is interested in how I am as a person and treats me individually*

| Completely agree |  | Agree |  | Neither agree nor disagree |  | Disagree |  | Completely disagree |
| --- | --- | --- | --- | --- | --- | --- | --- | --- |

*8. My physiotherapist identifies my physical and/or emotional status and adjusts the treatment according to the same*

| Completely agree |  | Agree |  | Neither agree nor disagree |  | Disagree |  | Completely disagree |
| --- | --- | --- | --- | --- | --- | --- | --- | --- |

*9. There is mutual trust between my physiotherapist and myself.*

| Completely agree |  | Agree |  | Neither agree nor disagree |  | Disagree |  | Completely disagree |
| --- | --- | --- | --- | --- | --- | --- | --- | --- |

*10. Between my physiotherapist and I there is a relationship based on respect.*

| Completely agree |  | Agree |  | Neither agree nor disagree |  | Disagree |  | Completely disagree |
| --- | --- | --- | --- | --- | --- | --- | --- | --- |

*11. My physiotherapist and I agree on what I want to achieve from the physiotherapy treatment.*

| Completely agree |  | Agree |  | Neither agree nor disagree |  | Disagree |  | Completely disagree |
| --- | --- | --- | --- | --- | --- | --- | --- | --- |

*12. My physiotherapist and I agree on which treatment to follow.*

| Completely agree |  | Agree |  | Neither agree nor disagree |  | Disagree |  | Completely disagree |
| --- | --- | --- | --- | --- | --- | --- | --- | --- |

*13. My physiotherapist knows perfectly well what he/she has to do.*

| Completely agree |  | Agree |  | Neither agree nor disagree |  | Disagree |  | Completely disagree |
| --- | --- | --- | --- | --- | --- | --- | --- | --- |

*14.*  *My physiotherapist performs his/her work with seriousness and honesty.*

| Completely agree |  | Agree |  | Neither agree nor disagree |  | Disagree |  | Completely disagree |
| --- | --- | --- | --- | --- | --- | --- | --- | --- |

*15. My physiotherapist informs me of my health problem.*

| Completely agree |  | Agree |  | Neither agree nor disagree |  | Disagree |  | Completely disagree |
| --- | --- | --- | --- | --- | --- | --- | --- | --- |

*16. My physiotherapist informs me of the physiotherapy treatment options for my problem.*

| Completely agree |  | Agree |  | Neither agree nor disagree |  | Disagree |  | Completely disagree |
| --- | --- | --- | --- | --- | --- | --- | --- | --- |

*17. When my physiotherapist explains exercises or health advice to me, he/she then asks about these and goes over them if necessary.*

| Completely disagree |  | Disagree |  | Neither agree nor disagree |  | Agree |  | Completely agree |
| --- | --- | --- | --- | --- | --- | --- | --- | --- |

*18. My physiotherapist makes me believe that I am able to get ahead with my own effort.*

| Completely disagree |  | Disagree |  | Neither agree nor disagree |  | Agree |  | Completely agree |
| --- | --- | --- | --- | --- | --- | --- | --- | --- |

*19. My physiotherapist makes me feel secure in what he says or does during the treatment process.*

| Completely disagree |  | Disagree |  | Neither agree nor disagree |  | Agree |  | Completely agree |
| --- | --- | --- | --- | --- | --- | --- | --- | --- |

*20. My physiotherapist understands how I feel.*

| Completely disagree |  | Disagree |  | Neither agree nor disagree |  | Agree |  | Completely agree |
| --- | --- | --- | --- | --- | --- | --- | --- | --- |

21. *My physiotherapist appears natural, sincere and honest at all times.*

| Completely disagree |  | Disagree |  | Neither agree nor disagree |  | Agree |  | Completely agree |
| --- | --- | --- | --- | --- | --- | --- | --- | --- |

*22. I feel that my physiotherapist accepts me as I am.*

| Completely disagree |  | Disagree |  | Neither agree nor disagree |  | Agree |  | Completely agree |
| --- | --- | --- | --- | --- | --- | --- | --- | --- |

*23. I observe a lack of coordination between the team of professionals (physiotherapists, doctors, aids, administration staff, etc.) who attend me.*

(Attention: if you are performing physiotherapy treatment at a center where only the physiotherapist works, mark the ‘Not applicable’ box)

| Completely disagree | Disagree | Neither agree nor disagree | Agree | Completely agree |  | Not applicable |  |  |  | Totalmente en desacuerdo |  | En desacuerdo |  | Ni de acuerdo ni en desacuerdo |  | De  acuerdo |  | Totalmente de acuerdo |
| --- | --- | --- | --- | --- | --- | --- | --- | --- | --- | --- | --- | --- | --- | --- | --- | --- | --- | --- |

*24. I feel that my physiotherapist has autonomy when making decisions about my treatment.*

| Completely disagree |  | Disagree |  | Neither agree nor disagree |  | Agree |  | Completely agree |
| --- | --- | --- | --- | --- | --- | --- | --- | --- |

*25. I feel that the space where the therapy takes place provides me privacy.*

| Completely disagree |  | Disagree |  | Neither agree nor disagree |  | Agree |  | Completely agree |
| --- | --- | --- | --- | --- | --- | --- | --- | --- |

*26. I feel that the words and gestures of my physiotherapist contradict each other.*

| Completely disagree |  | Disagree |  | Neither agree nor disagree |  | Agree |  | Completely agree |
| --- | --- | --- | --- | --- | --- | --- | --- | --- |

*27. The tone and volume of my physiotherapist’s voice generates trust.*

| Completely disagree |  | Disagree |  | Neither agree nor disagree |  | Agree |  | Completely agree |
| --- | --- | --- | --- | --- | --- | --- | --- | --- |

*28. My physiotherapist’s gaze generates trust.*

| Completely disagree |  | Disagree |  | Neither agree nor disagree |  | Agree |  | Completely agree |
| --- | --- | --- | --- | --- | --- | --- | --- | --- |

29. *I feel that my physiotherapist is interested in what I say.*

| Completely disagree |  | Disagree |  | Neither agree nor disagree |  | Agree |  | Completely agree |
| --- | --- | --- | --- | --- | --- | --- | --- | --- |

30. *My physiotherapist speaks to me in an easy and simple manner.*

| Completely disagree |  | Disagree |  | Neither agree nor disagree |  | Agree |  | Completely agree |
| --- | --- | --- | --- | --- | --- | --- | --- | --- |

*31. My physiotherapist knows how to express opinions opposed to mine, without making me feel bad.*

| Completely disagree |  | Disagree |  | Neither agree nor disagree |  | Agree |  | Completely agree |
| --- | --- | --- | --- | --- | --- | --- | --- | --- |

**THANK YOU VERY MUCH FOR YOUR COLLABORATION!**
