# Supplementary material for: Construction and content validation of a measurement tool to evaluate person-centered therapeutic relationships in physiotherapy services
Source: PLoS One. 2020 Mar 2;15(3):e0228916. doi: 10.1371/journal.pone.0228916 (PMC7051061; doi:10.1371/journal.pone.0228916)
Supplement: S1 Table — (PDF) [file pone.0228916.s004.pdf]

S1 Table. Delphi results

Round 1				Round 2				Round 3			
Item	% Agreement	Mean agreement	Nº suggestions for improvement	Item	% Agreement	Mean agreement	Nº suggestions for improvement	Item	% Agreement	Mean agreement	Nº suggestions for improvement
To what extent do you believe that your physiotherapist…? 1…seeks the positive side of what happens during your process of rehabilitation	55.5%	2.66	7	Item removed	--	--	--	--	--	--	--
2…makes you believe in your capabilities to get ahead with your effort	88.8%	3.67	3	1… makes you believe in your capabilities to get ahead with your effort	100%	3.89	0	1 … makes you believe in your capabilities to get ahead with your effort?	100%	3.89	0
3…demonstrates self-assurance in himself	88.8%	3.56	3	2…conveys assurance in what he/she tells you or says during the treatment process?	100%	3.89	0	2… conveys assurance in what he/she tells you or says during the treatment process?	100%	3.89	0
4…understands you	88.8%	3.11	6	3…understands how you feel and puts him/herself in your place	88,8%	3.45	1	3… understands how you feel and tries to put him/herself in your place?	100%	3.67	0
5…appears as he/she is at all time	88.8%	3.11	5	4…appears natural, sincere and honest at all times	100%	3.67	0	4…. appears natural, sincere and honest at all times?	100%	3.67	0
6…has judged you at any time	77.7%	3.44	4	5… has made you feel judged at any time	88.8%	3.56	1	5…has made you feel judged at any time?	100%	3.67	0

In your conversations with your physiotherapist, how often…?<br>7…do you notice that his/her gestures and his/her words say different things.	77.7%	3.33	5	6… do you notice that his/her body gestures, gaze and words are clear and not contradictory	100%	3.56	1	6… do you notice that his/her body gestures, gaze and words are clear and not contradictory?	100%	3.67	0
8…the tone of voice and the gestures of your physiotherapist generate confidence	100%	3.89	2	7…the expressions, tone and volume of the voice of your physiotherapist generate trust and proximity	100%	3.89	0	7… the expressions, tone and volume of the voice of your physiotherapist generate trust and proximity?	100%	3.89	0
9…does he/she look at you in your eyes when talking to you	77.7%	3.44	4	8…your therapist's gaze generates confidence and ease	100%	3.89	0	8… your therapist's gaze generates confidence and ease	100%	3.89	0
10…do you feel listened to	88.8%	3.56	4	9…do you feel that what you say is important for him/her and he/she tries to understand you	77,7%	3.22	2	9… do you feel that what you say is important for him/her and he/she tries to understand you?	100%	3.56	0
11…does he/she speak to you in a way that is easy for you to understand	100%	3.67	3	10…does he speak to you in an easy and simple manner	100%	3.89	1	10… does he speak to you in an easy and simple manner?	100%	3.89	0
				11…is he/she interested in knowing whether you have understood what he/she says?	100%	3.89	1	11… is he/she interested in knowing whether you have understood what he/she says?	100%	3.89	0
12…does he/she know how to tell you what he/she has to say without making you feel bad	88.8%	3.11	4	12…does he/she know how to tell you what he/she has to say, clearly and firmly, without making you feel bad.	88,8%	3.56	2	12…does he/she give you the necessary information, clearly and firmly, without making you feel bad?	100%	3.56	0
Regarding your treatment, how often do you consider that your physiotherapist…?,<br>13…knows perfectly well what	100%	3.67	4	13… knows perfectly well what	100%	4	0	13…. knows perfectly well	100%	4	0

he/she has to do				he/she has to do.				what he/she has to do?			
14…acts in the best possible way to improve your problem.	100%	3.89	3	14… acts in the best possible way to improve your problem.	100%	3.89	0	14…. acts in the best possible way to improve your problem?	100%	4	0
15…informs you of your problem and the treatment options	100%	3.67	4	15… informs you of your problem and the physiotherapy treatment options	100%	4	0	15 … informs you of your problem and the physiotherapy treatment options?	100%	4	0
16…after explaining exercises or care advice, later asks you about these and goes over them if necessary.	88.8%	3.67	3	16…after explaining exercises or care advice, later asks you about these and goes over them if necessary.	100%	3.67	1	16…. after explaining exercises or health advice, later asks you about these and goes over them if necessary.	100%	3.89	0
In your personal relationship with your physiotherapist, both during the treatment sessions as well as at any other time, indicate your degree of agreement regarding the following affirmations 17 I believe that my physiotherapist and I get on	100%	3.67	5	17 I believe that my physiotherapist and I have connected	88,8%	3.56	1	17 I believe that my physiotherapist and I have connected	100%	3.89	0
18 I feel cared for by my physiotherapist	100%	3.56	4	18 I feel that my physiotherapist provides me with the best possible care and attention	100%	4	0	18 I feel that my physiotherapist provides me with the best possible care and attention	100%	4	0
19 My physiotherapist is kind towards me	100%	3.78	0	19 My physiotherapist is kind towards me	100%	3.78	0	19 My physiotherapist is kind towards me	100%	3.89	0
20 I think I can count on my physiotherapist when I need it	77.7%	3.22	2	20 I think that my physiotherapist is an accessible person.	100%	3.89	0	20 I think that my physiotherapist is an accessible person.	100%	3.89	0
21.My physiotherapist shows interest and is involved in my problem	100%	3.89	4	21 My physiotherapist shows interest and concern for my problem	100%	4	0	21 My physiotherapist shows interest and concern for my problem.	100%	4	0
22.The treatment from my				22 The treatment from my				22 The treatment from			

physiotherapist makes me feel better	100%	3.89	1	physiotherapist makes me feel better emotionally	100%	3.89	0	my physiotherapist makes me feel better emotionally.	100%		0
During the treatment sessions, indicate to what degree you feel: 23. That your physiotherapist is interested in you as a person and treats you individually	100%	3.89	3	23 That your physiotherapist is interested in how you are as a person and treats you individually.	100%	3.89	1	23 That your physiotherapist is interested in how you are as a person and treats you individually.	100%	3.89	0
24. Your physiotherapist knows how to adapt to your physical and/or emotional status	88.8%	3.67	3	24 Your physiotherapist identifies your physical and/or emotional status and adjusts the treatment according to the same.	100%	3.89	0	24 Your physiotherapist identifies your physical and/or emotional status and adjusts the treatment according to the same.	100%	3.89	0
Indicate the degree to which you believe that between your physiotherapist and yourself: 25. There is mutual trust	100%	3.78	2	25. There is mutual trust	100%	4	0	25 There is mutual trust	100%	4	0
26. You respect one another	100%	3.89	2	26 There is a relationship based on respect.	100%	4	0	26 There is a relationship based on respect.	100%	4	0
27. You agree on the therapeutic objectives and the treatment	100%	3.67	3	27 You make a joint agreement on the therapeutic objectives and the treatment	100%	3.78	0	27 You make a joint agreement on the therapeutic objectives and the treatment.	100%	3.78	0
28. You collaborate to resolve the problems that may arise during your rehabilitation	88.8%	3.44	2	28 You collaborate to resolve together the problems that may arise during your rehabilitation	100%	3.89	0	28 You collaborate to resolve the problems that may arise during your rehabilitation	100%	3.89	0
Outside your relationship with your physiotherapist, you may have perceived some aspects concerning the organization of the service where your process of rehabilitation takes place. Indicate your level of agreement with these aspects:				29 I observe a lack of communication or coordination				29 I observe a lack of communication or			

29.I think there is a lack of communication among the team of professionals who attends me	100%	3.56	3	among the team of professionals who attends me.	100%	3.56	1	coordination among the team of professionals who attends me.	100%	3.78	0
30.I believe that my physiotherapist can perform his/her work with complete autonomy	100%	3.67	3	30 I feel that my therapist makes decisions independently regarding his/her treatment area	100%	3.78	0	30 I feel that my therapist makes decisions independently regarding his/her treatment area	100%	3.78	0
31. I feel that the space where the therapy takes place allows me to protect my privacy	100%	3.56	3	31 I feel that the space where the therapy takes place provides me privacy.	100%	3.89	0	31 I feel that the space where the therapy takes place provides me privacy.	100%	3.89	0
