# Supplementary material for: Construction and content validation of a measurement tool to evaluate person-centered therapeutic relationships in physiotherapy services
Source: PLoS One. 2020 Mar 2;15(3):e0228916. doi: 10.1371/journal.pone.0228916 (PMC7051061; doi:10.1371/journal.pone.0228916)
Supplement: S2 Table — (PDF) [file pone.0228916.s005.pdf]

S2 Table. Coding system for the analysis of cognitive interviews

CASM	Code	CATEGORY
COMPREHENSION	AC (APPROPRIATE COMPREHENSION)	The participant understands the meaning of the question
	IC (INAPPROPRIATE COMPREHENSION)	The participant does not understand or discusses the meaning of the question
	DC (DOUBTFUL COMPREHENSION)	The participant asks for clarification regarding the question
	PC (COMPREHENSION)	The participant has difficulty understanding the meaning of some words or concepts related to the question
	MC (MISTAKEN COMPREHENSION)	The participant understands the question in a different sense to what is meant
RETRIEVAL	ARE (APPROPRIATE RETRIEVAL)	The participant has no difficulty retrieving the necessary information for the question
	IRE (INAPPROPRIATE RETRIEVAL)	The participant has difficulty retrieving the necessary information for the response
REPORTING	IR (INAPPROPRIATE RESPONSE)	Inappropriate response or elaboration (incoherent with what the participant has understood or with the meaning that we meant to give to the question)
	ARC (APPROPRIATE RESPONSE CATEGOYR)	The final response fits in to one of the response categories given

	IRC (INAPPROPROIATE RESPONSE CATEGORY)	The final response does not fit in to one of the response categories
	DK (DOESN'T KNOW)	The participant does not know how to respond to the question
JUDGEMENT	IJ (INAPPROPRIATE JUDGEMENT)	The participant considers that he/she should not respond to the question (problems of social desirability, other problems)
INSTRUMENT LOGIC	RQ (REPEATED QUESTION)	The participant understands that the question is too similar to another, or that what is being asked is already contained in another item
	PO (PROBLEMS RELATED TO ORDER)	The order in which the item is placed within the questionnaire hampers understanding
	OTHER	Double-barreled question, double-negative questions, other problems.

[a]Behavior codes, extracted from *Using Behavior Coding to Evaluate the Effectiveness of Dependent Interviewing Joanne Pascale, US Census Bureau and Alice McGee, National Centre for Social Research*, in *PROCEEDINGS QUEST 2007 STATISTICS CANADA OTTAWA, ONTARIO CANADA APRIL 24th to 26th, 2007*

[b]Based on: Forsyth's O-Questionnaire Review Coding System combined *with Snijker's Expert Questionnaire Appraisal Coding System*
